# Investigating the impact of eight weeks of aerobic and resistance training on blood lipid profile in elderly with non-alcoholic fatty liver disease: a randomized clinical trial 

**Published:** 2019

**Authors:** Mohammad Ebrahim Ghamarchehreh, Alireza Shamsoddini, Seyed Moayed Alavian

**Affiliations:** 1 *Baqiyatallah Research Center for Gastroenterology and Liver Diseases, Baqiyatallah University of Medical Sciences, Tehran, Iran*; 2 *Exercise Physiology Research Center, Life Style Institute, Baqiyatallah University of Medical Sciences, Tehran, Iran*

**Keywords:** Aerobic training, Aging, Resistance training, Non-alcoholic fatty liver disease, Lipid profile

## Abstract

**Aim:**

The aim of this study was to investigate the effects of eight weeks aerobic and resistance exercise on blood lipid profile in elderly with non-alcoholic fatty liver disease (NAFLD).

**Background:**

Increased sedentary have a potential role in the development of NAFLD. Exercise training as an effective strategy to reduce NAFLD is presented.

**Methods:**

In a randomized clinical trial study, thirty nine elderly patients with NAFLD were enrolled and were randomly divided in three groups. Aerobic groups (AG, n=13), resistance group (RG, n=13) and control group (CG, n=13). AG participated in an 8-week aerobic training (three 45-min sessions per week at 55-75% of HRR (heart rate reserve)). RG participated in an 8-week resistance exercises (three 45-min sessions per week at 50-70% of 1RM (one-repetition measure). Blood lipid profile of patients were evaluated baseline and after eight weeks.

**Results:**

At baseline, there were no differences between the two groups. In two group, cholesterol and low density lipoprotein (LDL) of blood of elderly follow eight weeks aerobic training with p=0.02 and p=0.02 were decreased, respectively. Also, High density lipoprotein (HDL) was improved follow aerobic training (p=0.008). However, the aerobic and resistance training were not effective on triglyceride (TG).

**Conclusion:**

aerobic training was more effective than resistance training in improving the blood lipid profile in elderly with NAFLD and can role in management of this condition.

## Introduction

 Non-alcoholic fatty liver disease (NAFLD) is the disease of human liver cells which has been increasingly raised during the recent years due to sedentary and increased obesity in human societies as well as all advanced and developing countries. Currently, the most prevalent liver disease is NAFLD (1, 2). Prevalence of this disease is reported one out of five persons to one out of three persons across the different countries and based on the various diagnosis criteria (3). A review article has reported that prevalence of mild, moderate and severe fatty liver disease in Iran were estimated at 33.9%, 26.7% and 7.6%, respectively (4). In the same study, it was stated that NAFLD was more common among older men with obesity (4). NAFLD is prevalent among the elderly (5) and the biochemical, haematological and histological changes of fatty liver among the elderly are more severe than in the youth (6). Therefore, appropriate diagnosis and desired treatment of this disease are important tasks for clinical physicians, especially those dealing with the elderly (7). Prevalence of NAFLD in western societies and Asia countries is about 25-30% and 19-23%, respectively (8). In a study in Iran, the prevalence of NAFLD among the men was two times higher than the women (9). Due to acute and chronic problems resulting from NAFLD (10), it is highly necessary to investigate the ways in which the desired results are obtained without any medicine and in the least time. One medical aid approach and techniques taken into account and tried by scholars and specialists in recent years is the matter of conducting the physical activities and exercise training by people with NAFLD. Since the increased immobility can play a potential role in expansion of NAFLD . 

The physical activities and exercise training in turn, can be seen as a solution to treatment of this disease (11, 12). Changes in life style including the diet, losing weight, physical activity or exercise training are known as the first medical recommendations for treating the NAFLD , especially in absence of medicine (13, 14). Nowadays, performing the physical activities is discussed as an effective medical approach in the obesity reduction and appearance of NAFLD. Exercise training drives the energy inventories followed by active muscles. If the long-term exercise training comes with the negative energy balance, the size of adipose tissue will decrease. Hence, the studies indicate the impact of various activities and exercise training on treatment of some diseases such as NAFLD. Therefore, the present study aims at investigating the effect of aerobic and resistance exercise training on the blood lipid profile of elderly with non-alcoholic fatty liver. 

## Methods

**Patients and methods**

In a randomized clinical trial study, 39 old man patients with non-alcoholic fatty liver were selected from among those who had referred to the Fatty Liver Clinic and Digestion and Liver Clinics of Baqiyatallah Hospital of Tehran concerning the research conditions. Once the patients were introduced by the physician and in case of appropriate inclusion criteria and also to meet the ethical principles, participants completed the consent form and then they were verbally informed of how the study would be conducted. 

The exclusion right was given to patients in each temporal stage. Patients were randomly divided into three groups by Lottery: aerobic exercise (13 persons), resistance exercise (13 persons) and control group (13 persons). All the elderly participated in the study were evaluated in terms of heart and lung by the specialist. The inclusion criterion was liver fat over 5% as diagnosed by the sonography (15, 16). The exclusion criteria were: (1) patients under treatment due to increased sensitivity to insulin; (2) patients who are under the diet treatment; (3) patients who use alcohol; (4) patients with type 2 diabetes. 

In both exercise training groups (aerobic and resistance), patients participated in the 8-week follow-up program. But in the control group, the patients underwent the normal trend of treatment with this recommendation that during the 8-week aerobic and resistance exercise in both groups, the control subjects were not allowed to perform any exercise training.

**Anthropometric parameters and body composition**

Height was measured with wall-mounted stadiometer. Fat mass of whole body, Body Mass Index (BMI) and weight of patients were determined using the body composition analyzer (TANITA, TBF_300, Japan). Weight was measured without shoes and heavy clothes. 

**Aerobic exercise training program**

The aerobic exercise protocol was such that initially all patients started to warm up their bodies for the workout hall with moderate intensity. Then, each patient started to run on the treadmill for 15 minutes. Then patients took rest for 5 minutes and again continued running for 15 minutes. Finally, each patient cooled down for 5 minutes using the stretching exercise and smooth running. Exercise were performed for 8 weeks, each week with 3 non-consecutive sessions. Time of each exercise session was 45 minutes. Intensity of exercise during the activity period started from 55% of heart rate reserve and reached to 70%. The intensity increased every two weeks. The patients’ heart rate reserve were controlled by Polar heart rate monitors (17-20).

**Resistance exercise training program**

In the resistance group, patients warmed up their bodies for 10 minutes using the stretching exercise and running smoothly. Each patient began seven stationary movements in circular form. At the end, each patient cooled down the body for 5 minutes. To get familiar with the weightlifting technique and also adaptability with the environmental of exercise, the old participants conducted two sessions of exercise training. 

Seven resistance exercise performed circularly were: triceps press, biceps curl, calf raise, leg press, leg extension, lateral pull down and sit-ups. At the beginning of two weeks, a one-repetition measure (1RM) was measured. Intensity and number of movements in this research were such that initially the participants performed two periods with maximum 50 % of 1RM on the first and second weeks. On the third and fourth weeks, two periods were performed with 60% of on 1RM. On the fifth and sixth weeks, exercise was carried out with intensity the same as two previous weeks, but in three iterations. On the last two weeks, exercise was iterated with 70% of 1RM within three periods. On the first, second and third periods, 12, 10 and 8 iterations were performed. The rest time between the stations was 30 seconds and between each set (end of each period) was 90 seconds (8, 11, 21). 

**Biochemical testing and Outcome Variables**

Blood sampling was implemented from all subjects with the same conditions after a 12-14 hours, night fasting in two stages before the protocol implementation and 24 hours after protocol implementation. Seven milmol blood was drawn out of the brachial anterior vein while seated in each stage. Levels of total plasma cholesterol were measured by Calorimetric and Spanish kit of Biosystem. Triglyceride (TG) value was measured by enzyme colorimetry. Meanwhile, the plasma High-density lipoprotein (HDL) levels were measured by Calorimetric and Spanish kit of Biosystems. In this study, Low-density lipoprotein (LDL) of patients’ blood plasma was measured using the equation of Friedewald *et al.* (16). 

**Statistical analysis**

Descriptive statistics were used with means ± standard deviations (SD). To compare the mean of each dependant variables under study in three groups, one-way analysis of variance (ANOVA), independent t-test and dependant t-test as well as the Tukey’s Post Hoc test were used. Data were analysed by SPSS 20. P0.05 was considered significant. 

**Ethical considerations**

This study was approved by ethics committee of Baqiyatallah University of Medical Sciences (IR.BMSU.REC.1394.34). Also this study was approved in Iranian Registry of Clinical Trials (IRCTIRCT201701033871N1). Patients were (1) informed about the study's aims and procedures, (2) informed about the voluntary nature of their participation. 

## Results

**Diagram 1:**
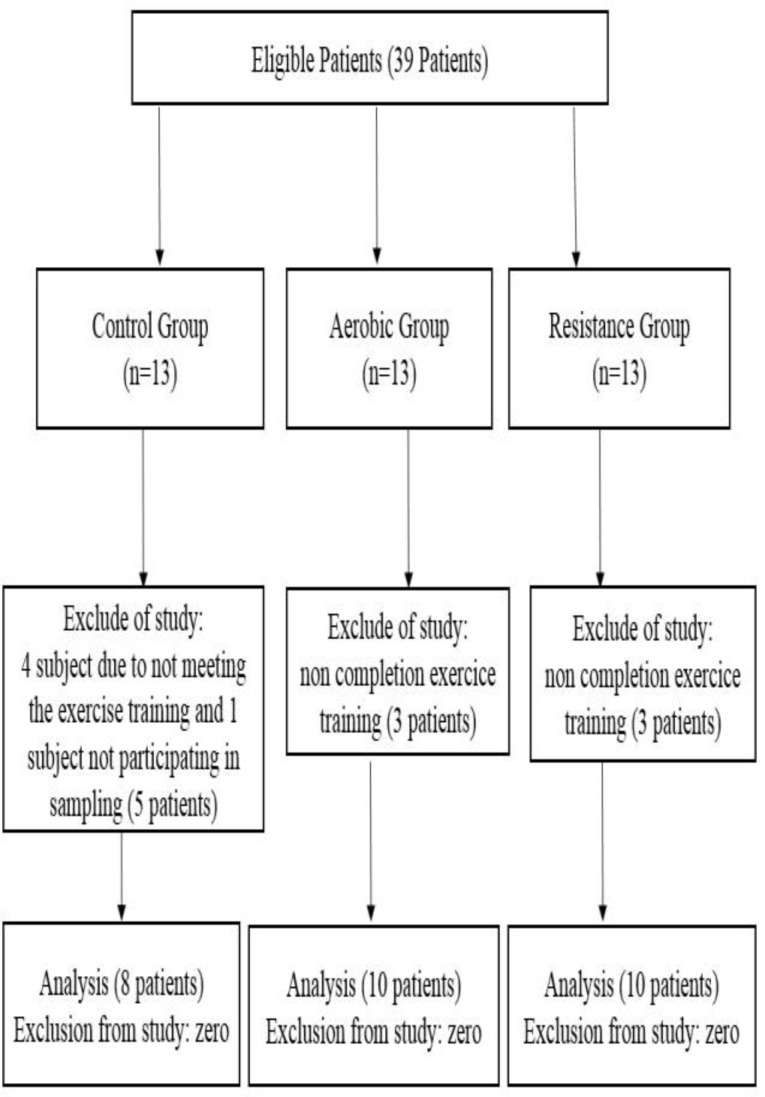
The CONSORT Flow Diagram of the sampling stages

Of the 39 patients enrolled in this study, 28 individuals completed the study protocol successfully. 5 patients from the control group (4 persons due to not meeting the exercise training and one person due to not completing the blood-sampling trend) and 6 patients from both treatment groups (due to no completing the exercise training) were excluded from the study (Diagram1). The anthropometrical characteristics of subjects in both groups are given in table 1. 

Results of statistical test of ANOVA showed that there is a significant difference in decreased blood cholesterol concentration among the groups. In this regard, results of post hoc test compared to the control group showed that the value of this variable in aerobic exercise group (P = 0.004) decreased significantly, but comparing the control group with the resistance exercise group (P=0.7), no significant decrease was observed. In comparing two treatment groups, a significant difference was observed between two exercise groups (P=0.02) (table 3). 

On the other hand, results of ANOVA indicated that there is no significant difference in reduction of TG concentration among three groups (P=0.62) (table 2). In this line, results of post hoc test showed that in comparison with the control group, value of this variable in aerobic exercise group (P=0.87) and resistance exercise group (P=0.88) had no significant reduction. There was no significant difference between aerobic and resistance exercise groups (P=0.6) (table 3).

In the present study, the results show that the HDL in comparison with the groups is observed to be statistically significant (P=0.007) (table 2). In this line, the post hoc test showed that compared to the control group, value of this variable in aerobic exercise group (P=0.008) increased significantly, but in comparing the control group with the resistance exercise group (P=0.82), there was no significant increase. In comparing two exercise groups, a significant difference was observed between them (P=0.03) (table 3). The results of ANOVA also suggested that there is a significant difference in decreased LDL among the groups (P=0.01) (table 2). In this line, results of post hoc test showed that compared to the control group, value of this variable in aerobic exercise group (P=0.02) decreased significantly, but in comparing the control group with the resistance exercise group (P=0.83), there was no significant decrease. In comparing both exercise groups, no significant decrease was observed (P=0.06) (table 3).

**Table 1:** Anthropometric Properties of the Participants in the Study

Variable	Control Group		Aerobic Group		Resistance Group	
	Baseline	After 8 week	Baseline	After 8 week	Baseline	After 8 week
Age	6.5±6.54		7.5±1.52		3.7±5.51	
High	4.4±172		8.2±8.174		7.8±9.174	
Weight	8.10±2.82	1.11±5.81	1.10±785.	7.11±9.83	3.16±3.94	1.16±2.92
BMI	3.4±2.28	9.3±5.27	1.3±1.28	5.3±5.27	6.2±6.30	6.2±9.29

**Table 2:** The lipid profile of the participants in this study

p	Control Group		Aerobic Group		Resistance Group		Variable
	After 8 week	Baseline	After 8 week	Baseline	After 8 week	Baseline	
62.0	8.103±6.184	3.161±9.207	1.19±7.140	8.21±178	8.20±1.152	6.17±1.162	TG (mg/dl)
004.0	7.33±1.200	4.30±7.200	10±1.137	7.13±2.213	4.12±2.174	1.13±2.182	Chol. (mg/dl)
007.0	2.5±6.38	7.4±8.37	5.2±6.43	2.2±4.38	2±9.38	2±3.37	HDL(mg/dl)
01.0	3.29±6.119	28±3.120	7±7.106	6.9±2.132	4.12±4.113	1.11±119	LDL(mg/dl)

**Table 3:** Post hoc test results in comparison of groups

TG		Chol		HDL		TG		Groups
p	AverageDifference	p	AverageDifference	p	AverageDifference	p	MeanDifference
87.0	14	004.0	1.40	008.0	4.4	02.0	8.24	Comparison of control group with aerobic group
88.0	3.13	76.0	8	82.0	8.0	83.0	8.4	Comparison of control group with Resistance group
6.0	27	02.0	1.32	03.0	6.3	06.0	9.19	Comparing aerobic group with Resistance group

## Discussion

Results showed that weight and BMI of subject in resistance group decreased significantly. That is while in the aerobic group, no significant decrease was observed in weight and BMI of subject. In control group, the mean of weight and BMI of subjects before and after the test showed no significant decrease. In the study conducted in The American College of Sports Medicine (ACSM), it was reported that aerobic exercise is more effective in creation of muscular hypertrophy among the sedentary people than the traditional resistance exercise (22). 

In our study, since the samples were inactive, it is likely that lack of weight loss in aerobic group is due to this fact. Therefore, the obtained result shows us fruitful and robust evidence indicating that regular and frequent aerobic exercise independent of weight loss has effective and useful impacts on improved lipid profile of people with fatty liver (23-25). Based on this, in the results of research group of the diabetes avoidance programs, it is reported that body activities and sport exercise are more effective than the weight loss (26). 

In accordance with the results, although the aerobic and resistance exercise reduce the plasma triglyceride, but such reduction is not significant. Studies have reported that only the exercise higher than the average level, intensity over 70% of maximum oxygen leads to decreased plasma triglyceride. This report states that in addition to exercise time, its intensity is also an important factor in workout plan (27). In this regards, it should be noted that our results are consistent with the ones reported by Alien de piano (21) and Johnson (18). These two studied have mentioned that the aerobic exercise reduces the plasma cholesterol, but not significantly. On the other hand, our results are not consistent with Gholami *et al*. (28) where it was revealed that the plasma cholesterol decreased significantly following eight weeks of aerobic exercise. 

Concerning the resistance exercise and plasma cholesterol, our results consistent with other studies (8) show that the resistance method has no significant impact on plasma cholesterol reduction among the people with NAFLD. Examining the effect of aerobic and resistance exercise on the total cholesterol in people with NAFLD , results indicated that only the aerobic exercise reduces the plasma cholesterol and resistance exercise has no impact. In this regard, our results are consistent with studies by Bae (29) and Gholami (28) and they all confirm the effect of aerobic exercise on plasma cholesterol. Although the results of resistance group are not in line with others’ results. Since the studies by Damor (8) and Zelbar-Saghi (30) have shown that the resistance exercise reduces the plasma cholesterol among the people with NAFLD. The important point is that the resistance exercise reduces the total plasma cholesterol, but why such results are not obtained in our study is likely due subjects’ inability in appropriate use of resistance exercise. Lack of weightlifting technique by the participants in our study could cause the person lift lighter weight and finally due to this matter, intensity of resistance exercise is not enough to decrease the cholesterol. 

Then like aerobic exercise, intensity of resistance exercise is also important in obtaining the given results. So, like the aerobic exercise in addition the number of movements, intensity of exercise, i.e. the amount of weights moved, is also important and effective in resistance exercise. 

Concerning the resistance exercise on the plasma HDL and LDL concentrations, it should be noted that only the aerobic exercise has a significant impact on HDL and LDL so that the plasma LDL is decreased and on other hand, the plasma HDL is increased, but the resistance exercise has not affected the plasma HDL and LDL. Ineffectiveness of resistance exercise on plasma HDL and LDL concentrations is likely resulting from the insufficient exercise which in turn, is due to the subjects’ inability in using the necessary technique to move the weights.

Studies have shown that exercise increases the activity of lipoprotein lipase and lecithin cholesterol transferase enzymes where these two enzymes reduce the total cholesterol, LDL, TG and raise HDL (31). Our results are consistent with previous literature to some extent and both are in the same line. In a study by Gholami *et al.,* which investigated the effect of aerobic exercise on the lipid profile of patients with fatty liver, it was reported that the aerobic exercise reduces the LDL and increases the HDL in patients with fatty liver (28). These results are in same direction with ours. In another study, Bae *et al.* reported that by performing the regular and fixed aerobic exercise among the patients with NAFLD, the total cholesterol and TG are decreased and HDL is increased (29). In the aerobic group, since we see the reduced fat percent of total body and also the fat reduction in the liver, such value in LDL is natural. Results showed that the aerobic exercise reduces the total cholesterol and LDL and increases the plasma HDL among the elderly with NAFLD. 

According to result of this study, the aerobic exercise seems to be more effective on the lipid profile of elderly with NAFLD than the resistance exercise and can play a more effective role in treating the fatty liver disease.

## Conflict of interests

The authors declare that they have no conflict of interest.

## References

[1] Smith BW, Adams LA (2011). Non-alcoholic fatty liver disease. Crit Rev Clin Lab Sci.

[2] Day CP (2006). Non-alcoholic fatty liver disease: current concepts and management strategies. Clin Med.

[3] Barshop N, Sirlin C, Schwimmer J, Lavine J (2008). Review article: epidemiology, pathogenesis and potential treatments of paediatric non‐alcoholic fatty liver disease. Aliment Pharmacol Ther.

[4] Moghaddasifar I, Lankarani KB, Moosazadeh M, Afshari M, Ghaemi A, Aliramezany M (2016). Prevalence of Non-alcoholic Fatty Liver Disease and Its Related Factors in Iran. Int J Organ Transplant Med.

[5] Mahady SE, George J (2016). Exercise and diet in the management of nonalcoholic fatty liver disease. Metabolism.

[6] Bertolotti M, Lonardo A, Mussi C, Baldelli E, Pellegrini E, Ballestri S (21). Nonalcoholic fatty liver disease and aging: epidemiology to management. World J Gastroenterol.

[7] Hamaguchi M, Kojima T, Ohbora A, Takeda N, Fukui M, Kato T (2012). Aging is a risk factor of nonalcoholic fatty liver disease in premenopausal women. World J Gastroenterol.

[8] Damor K, Mittal K, Bhalla AS, Sood R, Pandey RM, Guleria R (2014). Effect of progressive resistance exercise training on hepatic fat in Asian Indians with non-alcoholic fatty liver disease. British J Med Res.

[9] Sohrabpour AA, Rezvan H, Amini-Kafiabad S, Dayhim M, Merat S, Pourshams A (2010). Prevalence of nonalcoholic steatohepatitis in Iran: a population based study. Middle East J Dig Dis.

[10] Ghamar Chehreh ME, Vahedi M, Pourhoseingholi MA, Ashtari S, Khedmat H, Amin M (2013). Estimation of diagnosis and treatment costs of non-alcoholic Fatty liver disease: a two-year observation. Hepat Mon.

[11] Hallsworth K, Fattakhova G, Hollingsworth KG, Thoma C, Moore S, Taylor R (2011). Resistance exercise reduces liver fat and its mediators in non-alcoholic fatty liver disease independent of weight loss. Gut.

[12] Van der Windt DJ, Sud V, Zhang H, Tsung A, Huang H (2018). The Effects of Physical Exercise on Fatty Liver Disease. Gene Expr.

[13] Loria P, Adinolfi L, Bellentani S, Bugianesi E, Grieco A, Fargion S (2010). Practice guidelines for the diagnosis and management of nonalcoholic fatty liver disease: a decalogue from the Italian Association for the Study of the Liver (AISF) Expert Committee. Dig liver Dis.

[14] Shamsoddini A, Sobhani V, Ghamar Chehreh ME, Alavian SM, Zaree A (2015). Effect of Aerobic and Resistance Exercise Training on Liver Enzymes and Hepatic Fat in Iranian Men With Nonalcoholic Fatty Liver Disease. Hepat Mon.

[15] Szczepaniak LS, Nurenberg P, Leonard D, Browning JD, Reingold JS, Grundy S (2005). Magnetic resonance spectroscopy to measure hepatic triglyceride content: prevalence of hepatic steatosis in the general population. Am J Physiol Endocrinol Metab.

[16] Friedewald WT, Levy RI, Fredrickson DS (1972). Estimation of the concentration of low-density lipoprotein cholesterol in plasma, without use of the preparative ultracentrifuge. Clin Chem.

[17] Davoodi M, Moosavi H, Nikbakht M (2012). The effect of eight weeks selected aerobic exercise on liver parenchyma and liver enzymes (AST, ALT) of fat liver patients. J Shahrekord Univ Med Sci..

[18] Johnson NA, Sachinwalla T, Walton DW, Smith K, Armstrong A, Thompson MW (2009). Aerobic exercise training reduces hepatic and visceral lipids in obese individuals without weight loss. Hepatol.

[19] Devries MC, Samjoo IA, Hamadeh MJ, Tarnopolsky MA (2008). Effect of endurance exercise on hepatic lipid content, enzymes, and adiposity in men and women. Obesity.

[20] Haus JM, Solomon TP, Kelly KR, Fealy CE, Kullman EL, Scelsi AR (2013). Improved hepatic lipid composition following short-term exercise in nonalcoholic fatty liver disease. J Clin Endocrinol Metabol.

[21] De Piano A, de Mello MT, Sanches PdL, da Silva PL, Campos RM, Carnier J (2012). Long-term effects of aerobic plus resistance training on the adipokines and neuropeptides in nonalcoholic fatty liver disease obese adolescents. Europ J Gastroenterol Hepatol.

[22] Konopka AR, Harber MP (2014). Skeletal muscle hypertrophy after aerobic exercise training. Exerc Sport Sci Rev.

[23] Shaw K, Gennat H, O'Rourke P, Del Mar C (2006). Exercise for overweight or obesity. Cochrane Database Syst Rev.

[24] Hansen D, Dendale P, Berger J, Van Loon LJ, Meeusen R (2007). The effects of exercise training on fat-mass loss in obese patients during energy intake restriction. Sport Med.

[25] Franz MJ, VanWormer JJ, Crain AL, Boucher JL, Histon T, Caplan W (2007). Weight-loss outcomes: a systematic review and meta-analysis of weight-loss clinical trials with a minimum 1-year follow-up. J Am Diet Assoc.

[26] Group DPPR (2002). Reduction in the incidence of type 2 diabetes with lifestyle intervention or metformin. New England J Med.

[27] Sullivan S, Kirk EP, Mittendorfer B, Patterson BW, Klein S (2012). Randomized trial of exercise effect on intrahepatic triglyceride content and lipid kinetics in nonalcoholic fatty liver disease. Hepatol.

[28] Gholami N, Salekzamani Y, Zareh Nahandi M, Sokhtehzari S, Monazami AH, Rostami Nejad M (2013). The effect of aerobic exercise on serum level of liver enzymes and liver echogenicity in patients with non alcoholic fatty liver disease. Gastroenterol Hepatol Bed Bench.

[29] Bae JC, Suh S, Park SE, Rhee EJ, Park CY, Oh KW (2012). Regular exercise is associated with a reduction in the risk of NAFLD and decreased liver enzymes in individuals with NAFLD independent of obesity in Korean adults. PLoS One.

[30] Zelber-Sagi S, Buch A, Yeshua H, Vaisman N, Webb M, Harari G (2014). Effect of resistance training on non-alcoholic fatty-liver disease a randomized-clinical trial. WJG.

[31] Sugiura H, Sugiura H, Kajima K, Mirbod SM, Iwata H, Matsuoka T (2002). Effects of long-term moderate exercise and increase in number of daily steps on serum lipids in women: randomised controlled trial [ISRCTN21921919]. BMC Womens Health.

